# Elucidating the changes in the heterogeneity and function of radiation-induced cardiac macrophages using single-cell RNA sequencing

**DOI:** 10.3389/fimmu.2024.1363278

**Published:** 2024-03-27

**Authors:** Chunxiang Cao, Ran Wu, Shubei Wang, Lingfang Zhuang, Peizhan Chen, Shuyan Li, Qian Zhu, Huan Li, Yingying Lin, Min Li, Lu Cao, Jiayi Chen

**Affiliations:** ^1^ Department of Radiation Oncology, Ruijin Hospital, Shanghai Jiao Tong University School of Medicine, Shanghai, China; ^2^ Shanghai Key Laboratory of Proton-therapy, Shanghai, China; ^3^ Department of Cardiovascular Medicine, Ruijin Hospital, Shanghai Jiao Tong University School of Medicine, Shanghai, China; ^4^ Clinical Research Center, Ruijin Hospital, Shanghai Jiao Tong University School of Medicine, Shanghai, China

**Keywords:** macrophage, irradiation, heart injury, single-cell RNA sequencing, senescence, interferon, DNA damage, mitochondrial dysfunction

## Abstract

**Purpose:**

A mouse model of irradiation (IR)-induced heart injury was established to investigate the early changes in cardiac function after radiation and the role of cardiac macrophages in this process.

**Methods:**

Cardiac function was evaluated by heart-to-tibia ratio, lung-to-heart ratio and echocardiography. Immunofluorescence staining and flow cytometry analysis were used to evaluate the changes of macrophages in the heart. Immune cells from heart tissues were sorted by magnetic beads for single-cell RNA sequencing, and the subsets of macrophages were identified and analyzed. Trajectory analysis was used to explore the differentiation relationship of each macrophage subset. The differentially expressed genes (DEGs) were compared, and the related enriched pathways were identified. Single-cell regulatory network inference and clustering (SCENIC) analysis was performed to identify the potential transcription factors (TFs) which participated in this process.

**Results:**

Cardiac function temporarily decreased on Day 7 and returned to normal level on Day 35, accompanied by macrophages decreased and increased respectively. Then, we identified 7 clusters of macrophages by single-cell RNA sequencing and found two kinds of stage specific macrophages: senescence-associated macrophage (Cdkn1a^high^C5ar1^high^) on Day 7 and interferon-associated macrophage (Ccr2^high^Isg15^high^) on Day 35. Moreover, we observed cardiac macrophages polarized over these two-time points based on M1/M2 and CCR2/major histocompatibility complex II (MHCII) expression. Finally, Kyoto Encyclopedia of Genes and Genomes (KEGG) and Gene Ontology (GO) enrichment analyses suggested that macrophages on Day 7 were characterized by an inflammatory senescent phenotype with enhanced chemotaxis and inflammatory factors, while macrophages on Day 35 showed enhanced phagocytosis with reduced inflammation, which was associated with interferon-related pathways. SCENIC analysis showed AP-1 family members were associated with IR-induced macrophages changes.

**Conclusion:**

We are the first study to characterize the diversity, features, and evolution of macrophages during the early stages in an IR-induced cardiac injury animal model.

## Introduction

Radiation-induced cardiac events in breast cancer or lymphoma patients with relatively longer survival time have attracted much attention from researchers (1–4). In recent years, several studies have reported that incidental cardiac radiation is associated with cardiac morbidity and decreased survival in non-small cell lung cancer patients (5–8). Even with precise planning, some parts of the heart and the lung may still receive a radiation dose of more than 20 Gy in patients with thoracic tumors, leading to short and long-term effects (9).

Mónika Gabriella Kovács et al. found mild early-phase diastolic dysfunction at week one with decreased left ventricular weight in a Sprague−Dawley rat model (10). Tinna Christersdottir et al. showed that radiation-induced vascular inflammation persisted for years and suggested that early interleukin-1 blockade treatment may prevent inflammation (11). Some studies have also reported long-term persistent chronic inflammation following cardiac IR (12). These findings suggest that heart injury begins early and persists over time, which inspired us to explore early intervention strategies for IR-induced heart injury.

A healthy adult mouse’s heart contains all major leukocyte cell types (13), with macrophages being the largest among the non-myocardial cells at 8% (14, 15). Macrophages play key roles in tissue development, antigen presentation, inflammation resolution and tissue repair, particularly impacting cardiac remodeling, fibrosis, and cardiac dysfunction in disease models (15–18). Furthermore, the number and phenotype of macrophages are different depending on the disease condition (15). For example, inflammatory M1 macrophages emerge early after myocardial infarction (MI) and transform into anti-inflammatory and reparative M2 macrophages approximately one week after MI (19, 20). However, this classification was based on *in vitro* stimulation by specific stimuli. In fact, macrophage polarization *in vivo* is a dynamic process, and its role at different stages is oversimplified as either inflammatory or anti-inflammatory (15). Recent studies using single-cell RNA sequencing have revealed a more nuanced understanding on diversity and dynamics of macrophages, shedding light on their role in in the progression of heart disease (21–26).

Macrophages have been reported to expand in healthy tissues after IR, such as alveoli, human arteries and animal hearts (11, 27–29). However, the specific role of macrophages in radiation-induced heart injury isn’t fully understood. In our present research, we used a IR-induced heart injury model and found early changes in heart function accompanied by changing in macrophages. Then, we utilized magnetic beads to sort all cardiac immune cells and analyzed macrophages by single-cell RNA sequencing. To our knowledge, this is the first study to describe the heterogeneity, diversity and dynamics of cardiac macrophages in early stage of IR-induced heart injury.

## Materials and methods

### Animal care and IR procedure

Adult male C57BL/6J mice aged 7-9 weeks (20-25 g) were randomly assigned to the cardiac IR or sham IR group. The mice were housed in cages with a 12-hour light-dark cycle and fed standard laboratory chow and water ad libitum.

In IR group, mice heart received a single-dose IR of 20 Gy according to the dose used in previous studies (28, 30, 31). As described before, the mice were placed in a vertical position and received IR locally to the heart by a VARIAN linear accelerator (32).

### Echocardiography

Echocardiography was performed in mice under isoflurane anesthesia using a vevo-LAZR-X system. Hair was removed from the anterior chest using hair removal cream. Ultrasound gel was applied to the chest. Two-dimensional long-Axis M-Mode images were used to evaluate left ventricular (LV) systolic function. The trans-mitral inflow pattern and tissue doppler were used in the modified 4-chamber apical view to evaluate LV diastolic function. Analyses were performed blinded to the treatment group, and three images from consecutive cardiac cycles were analyzed and averaged.

### Tissue harvest

For the flow cytometry experiments, hearts were perfused through the left ventricle with 10 ml ice-cold PBS, excised, minced into small pieces (1-3 mm^3^) and subjected to enzymatic digestion with 500 U/ml collagenase II and 60 U/ml DNase I (all Worthington) for 1 hour at 37°C under gentle agitation. For the immunofluorescence experiments, hearts were perfused with 10 ml 4% paraformaldehyde after PBS perfusion, excised and fixed in 4% paraformaldehyde overnight. To obtain the lung to heart weight ratio, lungs and hearts were excised and weighed after perfusion with PBS. To obtain the heart-to-tibia ratio, each right tibia was bluntly isolated and measured by a Vernier caliper.

### Immunofluorescence staining

Each heart was incised transversely along the lower edge of the atrium, embedded with paraffin and cut into 5-µm-thick slices. The sections were dewaxed, rehydrated and blocked with 0.1% (v/v) Triton X-100/0.25% bovine serum albumin (BSA), and were subsequently incubated with various primary antibodies, fluorescently-labeled secondary antibodies and 4, 6-diamidino-2-phenylindole (DAPI, Solarbio, D8200). The following primary antibodies were used: rabbit anti-cardiac troponin T (CTNT, Abcam, ab209813, 1:500), and rabbit anti-CD68 (Boster, BA3638, 1:200). CY3-conjugated anti-rabbit secondary antibody (Abcam, ab6939, 1:200, anti-CD68), or CY5-conjugated anti-rabbit secondary antibody (Abcam, ab6564, 1:500, anti-CTNT) was applied following primary antibody incubation. The images were observed under a fluorescence microscope (Nikon, ECLIPSE 80I). Six 40× fields were randomly selected and taken photos to count the average number of positive cells.

### γH2AX apoptosis assay

One hour after IR or sham treatment, mouse hearts were removed, embedded and sectioned for immunofluorescence staining of phosphorylated histone H2AX (γH2AX), a marker of double-stranded DNA breaks (33, 34). In brief, the sections were prepared, dewaxed, and washed in PBS. Then, antigen retrieval was performed by boiling the sections in a microwave oven with an antigen retrieval solution. Next, the sections were blocked in 8% BSA and washed with PBS for 5 min before the buffer was removed. Then, 100 microliters of γH2AX antibody (Abcam, ab81299) diluted 1:1000 was added to each section overnight at 4°C. Sections were then washed and incubated with CY-conjugated anti-rabbit secondary antibody (Abcam, ab6939, 1:200) for 50 mins at room temperature. Finally, the slides were rinsed with PBS three times for 5 mins each and stained with DAPI at room temperature for 10 mins. The images were collected under a fluorescence microscope (Nikon, ECLIPSE 80I).

### Flow cytometry

After digestion, the single-cell suspension was generated and filtered through a 40 µm preseparation column, centrifuged at 400×g for 5 mins, and resuspended in PBS. A Zombie Aqua™ Fixable Viability Kit (Biolegend, 423101) was used to identify live cells for 20 min in the dark according to the manufacturer’s instructions. After that, the suspension was centrifuged again at 400×g for 5 mins and resuspended in stain buffer (BD Biosciences, 554657). Purified anti-mouse CD16/32 (Biolegend, 101301) was used to block Fc of the cells on ice for 5 min, and then the cells were stained at 4°C for 30 min with the following antibodies: anti-mouse CD45-APC/750 (Biolegend, 103154), anti-mouse CD11b-FITC (Biolegend, 101206), anti-mouse Ly6G-PE/Cy7 (Biolegend, 127618), anti-mouse F4/80-APC (Biolegend, 123116), anti-mouse Ly6C-PE (Biolegend, 128007), anti-mouse I-A/I-E-BV605 (Biolegend, 107639), anti-mouse CD206-BV421 (Biolegend, 141717), or anti-mouse C-C motif chemokine receptor 2 (CCR2)-BV421 (Biolegend, 150605). Next, stain buffer was added, and the cells were washed twice. Flow cytometry analysis was performed on the second day, and the data were analyzed with FlowJo software.

### Single-cell library preparation and sequencing

After digestion, six single cell suspensions in each group were mixed into one sample and incubated with CD45 MicroBeads (Miltenyi Biotec, 130-052-301) for 10 mins at 4°C, and isolated by a MACS Separator (Miltenyi Biotec) according to the manufacturer’s instructions. Single Cell 3’ kit v3 (10× Genomics) was used to form Gel Beads-in-emulsion (GEMs) and generate libraries from the DNA molecules and 10× barcodes. Cells were loaded onto a microwell chip and the RNA was extracted, reverse-transcribed to cDNA and amplified for 14 cycles. Sequencing was performed on the NextSeq 6000 Illumina sequencing platform following 10× Genomics instructions. The detected raw reads were mapped to the mouse genome (mm10) based on the number of barcodes and unique molecular identifiers (UMI) using the CellRanger software pipeline (v3.1.0) provided by 10× Genomics. The process above were supported of by OE Biotech. Co., Ltd. (Shanghai, China). We then processed the UMI count matrix by using the R package Seurat (version 4.0).

### Quality control of single-cell RNA sequencing data

To remove low-quality cells and possible multiple captures, we applied a criterion to filter out cells with UMI/gene numbers outside the limit of ±2 standard deviations of the mean, assuming a Gaussian distribution for the number of UMI/genes per cell. After visual inspection of the distribution of cells by the proportion of expressed mitochondrial genes, we further discarded low-quality cells with a certain percentage of counts belonging to mitochondrial genes. Library size normalization was performed on the filtered matrix in Seurat to obtain normalized counts.

### Differentially expressed genes and pathway enrichment analysis

Average expression and dispersion were calculated for each gene, and genes were then placed into multiple clusters based on expression. The batch effect of single cell expression profile data was corrected using the mutual nearest neighbors (MNN) in the batchelor(version 1.6.3) package (35). MNN performs dimensionality reduction on the logarithmic transformation of the gene barcode matrix. The MNN result is visualized in two-dimensional space by t-SNE (t-distributed stochastic neighbor embedding) (nonlinear dimensionality reduction).

The Seurat (36) package was used to identify DEGs. *P* value < 0.05 and |log2foldchange| > 1.5were set as the thresholds for significantly differential expression. DEGs were visualized in the form of heatmaps or volcano maps and used as input datasets for further GO or KEGG pathway analyses. Representative GO terms were selected from the top 30 terms, and KEGG terms were selected from the top 20 terms. All of them had a *P* value < 0.05.

### Pesudotime trajectory analysis

Pseudotime analysis was performed on the macrophage subclusters and two monocyte subclusters using the Monocle2 R package (v2.9.0) (37). In brief, the importCDS function of the Monocle2 package was used to convert the Seurat object into the CellDataSet object, and the differalGeneTest function was used to screen out the genes for sorting cells (q-value < 0.01). Then, the reduceDimension function was used for dimensionality reduction clustering. Finally, the orderCells function was used to infer the differentiation trajectory.

### Cell score and signature analysis

The AddModuleScore function in the Seurat R package was used to define the cell scores. Specifically, the AddModuleScore function calculated the average expression levels of each cluster at the single-cell level, subtracted by the aggregated expression of control feature sets. All analyzed features were binned based on averaged expression, and the control features were randomly selected from each bin. To assign M1/M2 polarization and pro-/anti-inflammatory ability, we used the average expression of genes previously used by Yunfan Sun et al. to define the M1/M2 scores and pro-/anti-inflammatory scores for each macrophage subcluster (38). For the MHCII score, we referred to the MHCII gene list reported in Elisa Martini’s article (25). Of note, we combined “Fabp4” and genes expressed in IR-induced senescent macrophages as a collection of senescent genes (39). The average expression of genes used by Zhuang Lingfang et al. was utilized to define chemotaxis, phagocytosis, oxidative phosphorylation (OXPHOS), glycolysis, fatty acid oxidation (FAO) and myeloid-derived suppressor cell (MDSC) scores for macrophage subclusters (24). Similarly, gene scores by groups were also performed according to the gene sets described above. In addition, genes referred to in previous studies that were not expressed in our study were excluded. The results are shown by violin plots or bubble charts.

### Single-cell regulatory network inference and clustering analysis

RcisTarget’s motif database and GRNboost (SCENIC v1.1.2.2, RcisTarget v1.2.1 and AUCell v1.4.1) (40)were run with default parameters. First, predicted target genes for each TFwere identified based on coexpression. Then, the transcription factors identified from the data and their corresponding target genes were identified based on motif analysis by the RcisTarget package. Finally, the activity of each regulator in each cell was scored by the AUCell package.

Additionally, we tried to identify coexpressed modules (regulon) between and potential target genes (41), as well as the regulon activity score (RAS) of each group; regulon specificity score (RSS) was calculated to obtain the specific correspondence between the predicted regulon and each group. The connection specificity index (CSI) of regulon was used to indicate the correlation between different regulons. Regulons with high CSI might co-regulate downstream genes and be responsible for cell functions.

### Quantitative real-time polymerase chain reaction

After single-cell RNA sequencing, the total RNA of the remaining immune cells from each sample was extracted with invitrogen TRIzol® Reagent. cDNA was synthesized with HiScript® III RT SuperMix for qPCR (+gDNA wiper) (Vazyme Biotech Co.,Ltd., Nanjing, China) according to the protocol provided by the manufacturer. The primers for genes of interest were designed and synthesized by sangon biotech Co.,Ltd. (Shanghai China) and the sequences are shown in **Supplementary Table 1**. All reactions were performed at least three times for every sample. PCR ampliffcation was performed using ChamQ SYBR qPCR Master Mix (High ROX Premixed) (Vazyme Biotech Co.,Ltd., Nanjing, China). All the relative mRNA expression levels were normalized to the level of β-actin in each sample.

### Statistics

All statistical analyses were conducted with GraphPad Prism software. Data are presented as the mean ± SD. Two group comparisons were analyzed by the unpaired t test. More than two group comparisons were analyzed using one-way ANOVA. The Shapiro−Wilk (SW) test or Anderson−Darling (AD) test was performed to assess normal distribution. If the SW or AD test passed, a 2-tailed unpaired Student’s t-test (two groups) or ANOVA test (more than two groups) was used, followed by Tukey’s multiple comparisons test when the Brown-Forsythe test was passed or the Welch test when the Brown-Forsythe test did not pass. If the SW test or AD test did not pass, the Mann–Whitney test (two groups) or nonparametric Kruskal–Wallis test (more than two groups) was used followed by Dunn’s multiple comparisons test. Statistical significance was considered at *P<0.05, **P<0.01, ***P<0.001, and **** *P*<0.0001.

## Results

### Cardiac injury after IR is accompanied by macrophage changes

**Figure 1:**
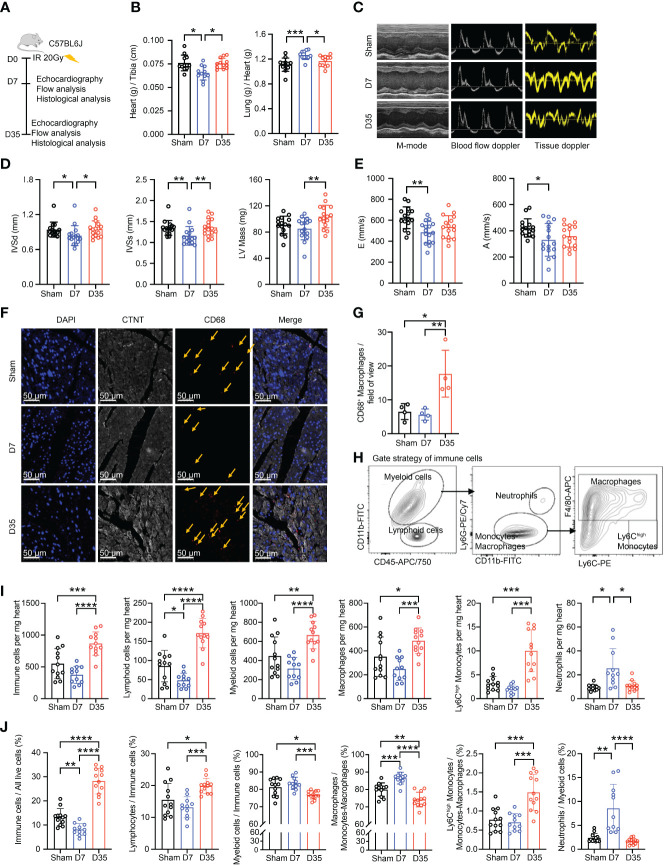
Transient changes in cardiac function are accompanied by changes in the number of cardiac macrophages. **(A)** Schematic diagram of animal modeling and experimental procedures. **(B)** Comparisons on Heart (g) to Tibia (cm) ratio^a^ and Lung (g) to Heart (g) ratio^b^ (Sham, n=12; D7, n=11; D35, n=12). Data were pooled from three independent experiments. **(C)** Representative images of echocardiography in cardiac function. M-mode, blood flow doppler and tissue doppler images views. (Sham, n=15; D7, n=16; D35, n=16). Data were pooled from three independent experiments. **(D)** Echocardiographic analysis of systolic function by M-mode: interventricular septum in diastole (IVSd^b^), interventricular septum in systole (IVSs^b^) and left ventricular (LV) Mass (corrected)^a^. **(E)** Echocardiographic analysis of diastolic function by blood flow doppler and tissue doppler: the speed E peak^a^ (left ventricular early-diastolic fast filling) and the speed A peak^a^ (left ventricular late-diastolic filling). **(F)** Representative images of immunofluorescent staining: CD68+ macrophages (Red), CTNT+ cardiomyocytes (Gray), DAPI+ nucleus (Blue). The yellow arrows point to macrophages. Scale bar, 50 μm. (n=4 in each group). **(G)** The statistical plot^a^ of panel **(F)**. **(H)** Flow cytometry gating scheme used to identify cardiac immune cells. **(I)** Comparison on number of immune cells in per mg heart tissue by flow analysis. Ly6C^high^ Monocytes^c^; Neutrophils^c^; the other cell types^a^. **(J)** Comparison on percentage of each cell type by flow analysis. Immune cells/All live cells (%)^c^; Ly6C^high^ Monocytes/Myeloid cells (%)^c^; Neutrophils/Myeloid cells (%)^b^; the other parameters^a^. Results of **(I, J)** were from three independent flow analyses. (Sham: n=12; D7: n=11; D35: n=12). “a”: Ordinary one-way ANOVA test; “b”: Kruskal-Wallis test; “c”: Welch ANOVA test. * *P*<0.05, ** *P*<0.01, *** *P*<0.001 and **** *P*<0.0001.

Male C57BL/6J mice were enrolled and irradiated with a single dose of 20 Gy or IR sham on the heart. Mice in IR group were killed one week or five weeks after IR, and mice in Sham group were killed one week after the sham operation. Echocardiography was performed just before the mice were sacrificed, and flow cytometry analysis or histological analysis was performed after sacrificing (**Figure 1A**).

First, we performed γH2AX immunofluorescence staining of heart tissue to confirm heart injury as reported in a previous study (34) (**Supplementary Figure S1A**). Second, we used the heart-to-tibia ratio and lung-to-heart ratio to evaluate cardiac morphology and function (**Figure 1B**). 7 days post-IR, the heart-to-tibia ratio was significantly reduced in the IR group compared with the Sham group. On Day 35, the heart-to-tibia ratio recovered to sham level. Opposite change was obtained for the lung-to-heart ratio. Above results indicated transient pulmonary congestion and a transient decrease in cardiac function on Day 7. Third, we utilized echocardiography to further evaluate cardiac function in mice (**Figure 1C**). For systolic function, the interventricular septum in diastole (IVSd) and interventricular septum in systole (IVSs) decreased significantly on Day 7, but there was no significant difference between Sham group and 35-day group. Moreover, the left ventricular (LV) mass (corrected) slightly decreased on Day 7 and apparently increased on Day 35 (**Figure 1D**). Left ventricular ejection fraction (LVEF) and left ventricular fractional shortening (LVFS), two main parameters of systolic function, decreased in the IR groups, but the differences were not significant. The other parameters of systolic function were not significant (**Supplementary Figure S1B**). In terms of cardiac diastolic function, the speed E peak of left ventricular early diastolic fast filling and the speed A peak of left ventricular late diastolic filling both decreased in the 7-day IR group (**Figure 1E**). However, the other diastolic parameters were not significantly changed among the groups (**Supplementary Figure S1C**).

Based on previous reports that macrophages accumulate in cardiac tissues after IR (27–29), we investigated the changes in macrophages in our mice model. Immunofluorescence staining showed a slight decrease in the number of macrophages in the 7-day group but a robust increase in the 35-day group, implying that macrophage number changes over time after IR (**Figures 1F, G**).

To further verify the changes in cardiac macrophages, cardiac immune cell flow cytometry was performed and analyzed using established gating strategies reported in other studies (18). Firstly, we distinguished live cells. Then, we defined CD45+ cells as immune cells, CD45+CD11b+ cells as myeloid cells, CD45+CD11b- cells as lymphoid cells, CD45+CD11b+Ly6G+ cells as neutrophils, CD45+CD11b+Ly6G- cells as monocytes/macrophages, CD45+CD11b+Ly6G-F4/80+ cells as macrophages and CD45+CD11b+Ly6G-F4/80-Ly6C^high^ cells as Ly6C^high^ monocytes (**Figure 1H**; **Supplementary Figure S1D**). We evaluated changes in immune cells, especially macrophages, in terms of both the absolute number and percentage of cells (**Figures 1I, J**). The numbers of cells per mg heart tissue, including total immune cells, lymphoid cells, myeloid cells, macrophages, and Ly6C^high^ monocytes, all decreased on Day 7 and significantly increased on Day 35. However, the highest number of neutrophils was present on Day 7. Moreover, the percentages of immune cells in live cells and lymphoid cells in immune cells decreased on Day 7 and increased on Day 35. While, the percentage of myeloid cells in immune cells was opposite to the lymphoid cells. Furthermore, the proportion of Ly6C^high^ monocytes in monocytes-macrophages robustly increased on Day 35. Lastly, the percentages of macrophages in monocytes-macrophages and neutrophils in myeloid cells both significantly peaked on Day 7 but declined on Day 35.

In summary, radiation-induced heart injury occurs one week after IR and is accompanied by changes in the immune microenvironment, especially macrophages. We suspect that this finding might indirectly reflect different early stages of IR-induced heart injury (13).

### Single-cell RNA sequencing identified cardiac macrophage subsets with distinct functions

To identify diverse macrophage subtypes and characterize their dynamic alterations during the early period after cardiac IR, we performed magnetic bead separation to collect live cardiac CD45+ leukocytes from the murine hearts in the Sham, 7-day and 35-day IR groups using a 10× Genomics Chromium platform and reagents (**Supplementary Figure S2A**). In addition, the cell suspensions from six hearts mixed as one sample in each group to reduce bias.

After quality trimming and filtering using the R package Seurat (version 4.0) (36), 26855 individual immune cells were included in downstream analyses. Unsupervised clustering and t-SNE dimensionality reduction implemented in the Seurat package were performed on this complex dataset (**Supplementary Figure S2B**). To remove the batch effects in single-cell RNA-sequencing data, the MNN were performed with the R package batchelor (42). We identified 5 clusters, including monocyte lineage (22356 cells), neutrophils (786 cells), basophils (82 cells), B cells (2384 cells) and T cells (1247 cells) (**Supplementary Figure S2C**). Each cell type was identifiable based on the significant expression of well-characterized marker genes (**Supplementary Figure S2D**).

Apparently, monocyte lineage was the most abundant immune cell type, accounting for more than 70% of cells in each group. The proportion of monocyte lineage gradually decreased after IR. The proportion of T and B cells apparently increased on Day 35. Specifically, neutrophil infiltration increased in the 7-day IR group compared with the Sham group, which was consistent with our flow cytometry results (**Figures 1I, J**; **Supplementary Figure S2C**).

**Figure 2:**
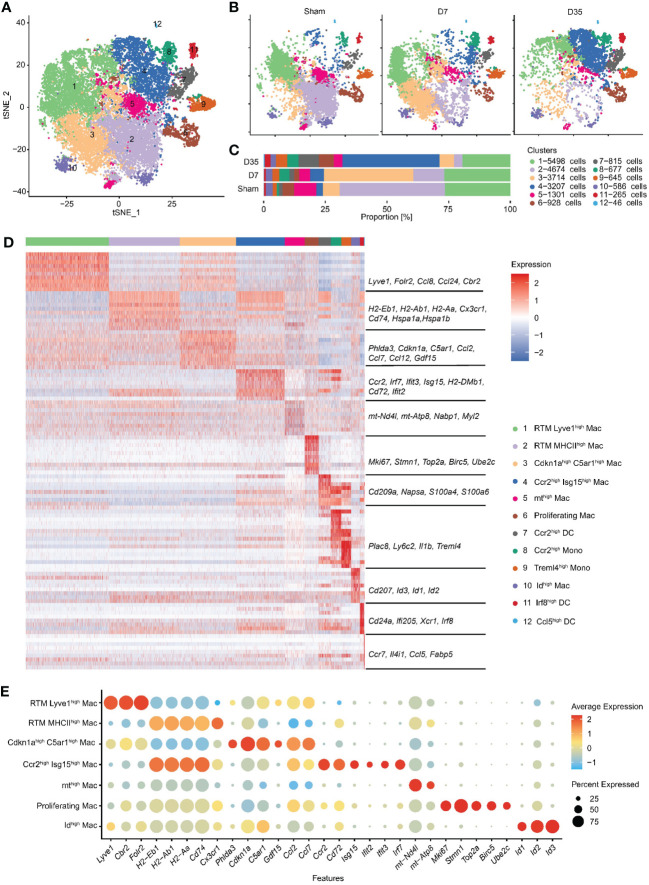
Second-level clustering analysis on monocyte lineage identified twelve distinct subclusters. **(A)** t-SNE dimensionality reduction analysis identified 12 subclusters based on monocyte lineage. **(B)** t-SNE represented 12 subclusters in Sham, D7 and D35 groups, respectively. **(C)** Bar plot showed the proportion of each subcluster differed among groups. **(D)** Heatmap showed top 10 differently expressed marker genes for each subcluster. The characteristic genes are listed in the middle, and the name of each cell cluster is on the right. **(E)** Bubble diagram showed the expression of characteristic genes for seven macrophage subsets. t-SNE, t-distributed stochastic neighbor embedding; DC, dendritic cell.

To learn more about the heterogeneity and diversity of macrophages, we next performed second-level clustering analyses on the monocyte lineage. Seurat identified twelve distinct clusters (**Figure 2A**). The proportion of each cluster differed among groups. Cluster 1 and 2 were the two main cell types in the Sham group, but Cluster 3 and 4 became the main cell type in the 7-day and 35-day groups, respectively (**Figures 2B, C**). Finally, seven macrophage clusters (Clusters 1, 2, 3, 4, 5, 6, and 10), two monocyte clusters (Clusters 8 and 9) and three dendritic cell (DC) clusters (Clusters 7, 11, and 12) were identified. They emerged each with a unique transcriptional profile (**Figure 2D**).

We used bubble charts to visualize the characteristic genes of each macrophage subpopulation (**Figure 2E**). In detail, Cluster 1 and 3 both expressed classic resident macrophage (RTM) markers (Lyve1, Cbr2, Folr2). Cluster 1, which expressed higher RTM markers, was similar to the previously described Timd4+ or Folr2+ cluster (21, 24). In contrast, Cluster 3 highly expressed inflammation-related genes (Ccl2, Ccl7, Ccl12), endoplasmic reticulum stress-related genes (Phlda3 and C5ar1) (43, 44), mitochondrial metabolism-related marker (Gdf15) (45), and cell cycle arrest and senescence biomarker (Cdkn1a) (46, 47). Therefore, we named Cluster 1 RTM Lyve1^high^ Mac and named Cluster 3 Cdkn1a^high^C5ar1^high^ Mac based on their characteristic genes.

Next, Cluster 2 and Cluster 4 both had higher expression of antigen-presentation genes (MHCII, such as H2-Eb1, H2-Ab1, H2-Aa, CD74). Specifically, Cluster 2 expressed the RTM marker Cx3cr1, and heat shock protein genes (Hspa1a, Hspa1b) which are related to protein folding (48). Cluster 4 highly expressed Ccr2, Cd72 and interferon-related genes (Isg15, Ifit2, Ifit3, Irf7). Thus, we termed Cluster 2 RTM MHCII^high^ Mac and Cluster 4 Ccr2^high^ Isg15^high^ Mac.

The remaining three macrophage subsets each accounted for a very small proportion of all macrophages. We named Cluster 5 and Cluster 10 mt^high^ Mac and Id^high^ Mac respectively based on their own higher expressed genes. Cluster 6 was identified as “Proliferating Mac” with highly expressed cell cycle and proliferation markers (Stmn1, Top2a, Birc5, and Ube2c) (49). Finally, we identified two monocyte clusters as Ccr2^high^ Mono (Cluster 8) and Treml4^high^ Mono (Cluster 9) and three DC clusters as Ccr2^high^ DCs, Irf8^high^ DCs, and Ccl5^high^ DCs (Clusters 7, 11, and 12) (**Figure 2D**).

In summary, we identified twelve subclusters on the monocyte lineage, and observed two RTM subsets and two stage-specific macrophage subsets in our animal model.

### Temporal dynamics of monocytes and macrophages by pseudotime trajectory analysis

**Figure 3:**
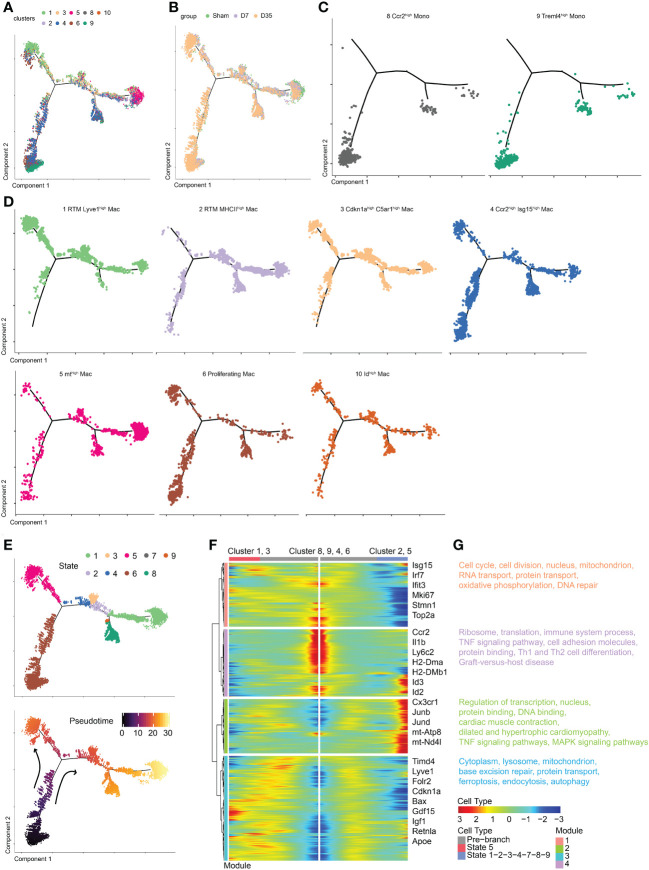
Temporal dynamics of monocytes and macrophages in Sham and IR heart tissues by pseudotime trajectory analysis. **(A)** t-SNE visualization of cells arranged along trajectories, colored by identified subset. **(B)** Visualization of cardiac macrophage trajectories split by samples. **(C, D)** Visualization of two monocyte subsets and seven macrophage subsets trajectories split. **(E)** Monocle 2 pseudotime trajectory analysis for manually evaluated subclusters of monocytes and macrophages, color coded by state (up) and pseudotime (down). **(F)** Heatmap showing the dynamic changes in gene expression along the pseudotime. **(G)** Selected top GO and KEEG terms of each module. t-SNE, t-distributed stochastic neighbor embedding; KEGG, the Kyoto Encyclopedia of Genes and Genomes; GO, Gene Ontology.

To determine the developmental relationship between monocytes and macrophage clusters, we performed pseudotime analysis to predict the trajectory of these cells over time (**Figure 3A**). Monocle 2 was applied to construct a developmental trajectory and superimposed Seurat-defined clusters on this trajectory. The cell developmental trajectories of the three groups are shown in **Figure 3B**. Two monocyte subclusters were considered as the starting cells for the analysis to predict monocyte development (**Figure 3C**). The trajectories of seven macrophage subclusters are shown in **Figure 3D**. We defined nine states based on the state trajectory analysis (**Figure 3E**).

Obviously, Cluster 1 and Cluster 3 shared a similar trajectory that seemed to be different from monocytes, and they went in the direction of state 5 (**Figures 3D, E**). Sarah A. Dick reported that RTMs with high expression of Lyve1 and Timd4 occupied a separate trajectory branch, which was in line with our Cluster 1 (21). Tissue cues may prompt recruited macrophages to ultimately express genes such as Timd4 and Lyve1 over time (21). The hypothesis is in agree with our trajectory analysis that state 5 (Cluster 1 and Cluster 3) was one endpoint of the trajectory branch. In addition, we hypothesized that Cluster 3 arose from Cluster 1 rather than from monocytes. These results also confirmed the idea that Lyve1-positive RTMs might self-proliferate.

Next, Cluster 2 and Cluster 5 occupied a second branch and went in the direction of state 1, which may be another trajectory endpoint (**Figures 3D, E**). Last, Clusters 4, 6 and 10 occupied the beginning branch and two other branches. They appeared to develop from monocytes, as their trajectories were continuous with monocytes. The trajectory of Cluster 4, which was enriched with Ccr2 and Isg15, was consistent with previous studies showing that CCR2+ macrophages probably arise from monocytes (21, 23). Gene expression was plotted as a function of pseudotime in Monocle to track gene expression changes across macrophage states (**Figure 3F**).

GO and KEGG pathway enrichments on each module were performed to further understand biological changes across different macrophage states (**Figure 3G**). In module 1, the pathways were associated with cell proliferation (cell cycle, cell division, DNA repair, nucleus), respiration (mitochondrion, oxidative phosphorylation), and RNA and protein transport. In module 2, we observed transcription- and translation-related (nucleus, regulation of transcription, protein and DNA binding), cardiac-related (cardiac muscle contraction, dilated and hypertrophic cardiomyopathy), and inflammatory-related (tumor necrosis factor (TNF) and MAPK signaling pathways) pathways. In module 3, base excision repair, protein transport, intracellular components (cytoplasm, lysosome, mitochondrion), and pathways associated with dead tissue elimination (ferroptosis, endocytosis, autophagy) were enriched. In module 4, ribosomes, translation, immune system processes, the TNF signaling pathway, cell adhesion molecules (CMAs) and pathways associated with protein binding, Th1 and Th2 cell differentiation, and graft-versus-host disease were enriched.

### Two IR-related macrophage subsets at different stages after cardiac IR

**Figure 4:**
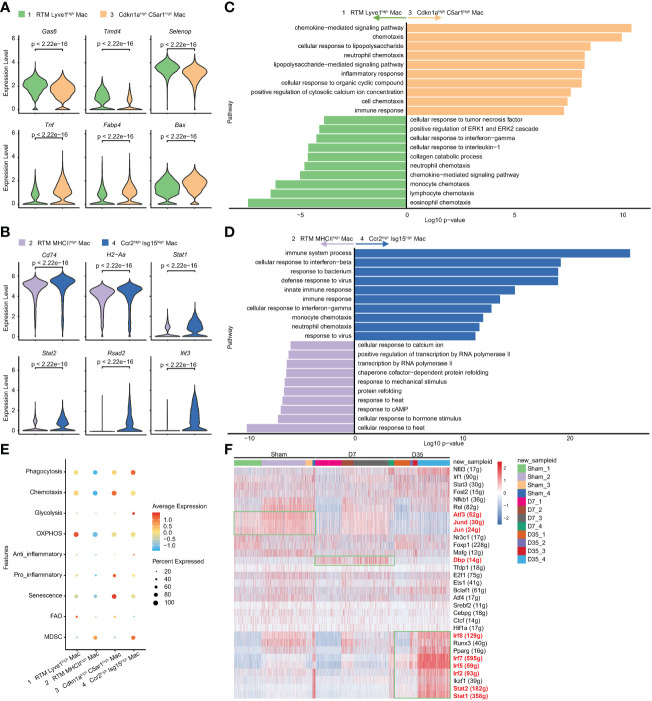
Characteristic genes and biological functions of two IR associated macrophages. **(A, B)** Violin plots of the characteristic gene expression: Cluster 3 vs Cluster 1 **(A)**; Cluster 4 vs Cluster 2 **(B)**. **(C, D)**. Top 10 terms on biological process by GO analysis: Cluster 3 vs Cluster 1 **(C)**; Cluster 4 vs Cluster 2 **(D)**. **(E)** Bubble diagram exhibiting scores of phagocytosis, chemotaxis, glycolysis, OXPHOS, anti-inflammatory, pro-inflammatory, senescence, FAO and MDSC in Cluster1-4. **(F)** Heatmap of transcriptional regulons among 4 macrophage subsets (Cluster1-4) in Sham, D7 and D35 groups. GO, Gene Ontology; OXPHOS, oxidative phosphorylation; FAO, fatty acid oxidation; MDSC, myeloid-derived suppressor cells.

To further study the two IR-related macrophages, we performed differential gene analysis and enrichment pathway analysis to compare them with their similar RTM clusters. The feature genes of Clusters 3 and 4 are shown in **Figures 4A, B**. In contrast with Cluster 1, the expression of classical RTM genes (Gas6, Timd4, Selenop) were lower, but inflammatory genes (Tnf and Fabp4) and apoptosis-related gene (Bax) were expressed at higher levels in Cluster 3. Cluster 4 was characterized by higher expression of MHCII genes (H2-Aa, Cd74) and interferon-related pathway genes (Stat1, Stat2, Rsad2, Ifit3), suggesting that Cluster 4 has different biological functions from Cluster 2.

The GO analysis showed that Cluster 3 was enriched in chemotaxis, inflammatory response, and chemokine-mediated signaling pathways, which indicating its inflammatory role. lipopolysaccharide (LPS) is usually used to induce macrophage polarization to the M1 subtype and induce inflammation (50). The LPS-related pathway might reflect the M1-like inflammatory role of Cluster 3 on Day 7 (**Figure 4C**). The KEGG enrichment analysis led to a similar conclusion: inflammatory-related signaling pathways (TNF, NF-kappa B, IL-17, Toll-like receptor), cytokine−cytokine receptor interaction and chemokine signaling pathway were enriched in Cluster 3 (**Supplementary Figure S3A**).

The comparative analysis was also performed between Cluster 2 and Cluster 4. GO analysis indicated that Cluster 4 showed more immune responses to bacteria and viruses and cellular responses to interferon-beta and interferon-gamma (**Figure 4D**). The KEGG analysis showed that phagosomes, CMAs, cytokine−cytokine receptor interactions, antigen processing and presentation (APP) and chemokine signaling pathways were enriched in Cluster 4 (**Supplementary Figure S3B**).

To understand the metabolic status and inflammatory status of the above four clusters of macrophages, we performed genetic scoring according to previous studies (**Figure 4E**) (24, 38). The related genes of each score are listed in the **Supplementary Table 2**. In particular, Fabp4, which is generally reported to be associated with metabolism and inflammation (51), was found to be related to aging (39). Lulu Su et al. reported the potential senescent genes expressed by macrophages in an IR-induced pulmonary fibrosis model (52). We gathered IR-induced senescent genes and “Fabp4” as our senescent gene set. The results showed that Cluster 1 had higher OXPHOS and FAO scores, which indicated that this subset consisted of lipid metabolism-associated macrophages. Cluster 3 had higher senescence, chemotaxis and pro-inflammatory scores, implying that it was associated with aging and inflammation. Cluster 4 had higher phagocytosis and anti-inflammatory scores, which was in line with our pathway enrichment results. Cluster 2 and Cluster 4 both had higher MDSC scores indicating their immunosuppressive function (53).

SCENIC analysis indicated that that the transcription factor activator protein-1 (AP-1) family members Jun and Jund, which could form heterodimeric complexes with the AP-1 family members activating transcription factor 3 (Atf3), were relatively overexpressed in Sham group especially in Cluster 2 and 3, and gradually decreased over time after heart IR. Moreover, D site albumin promoter binding protein (Dbp) was overexpressed in 7-day group especially in Cluster 1 and 3. Finally, Cluster 4 in 35-day group was rich in Stat1, Stat2, Irf2, Irf5, Irf7, and Irf8, which were all associated with interferon-related biological processes (**Figure 4F**).

In summary, Cluster 3 on Day 7 was a senescence-related macrophage with enhanced chemotaxis and inflammatory function, while Cluster 4 on Day 35 was an interferon-related macrophage with better phagocytosis and APP ability.

### Polarization and dynamics of cardiac macrophage over time after IR

**Figure 5:**
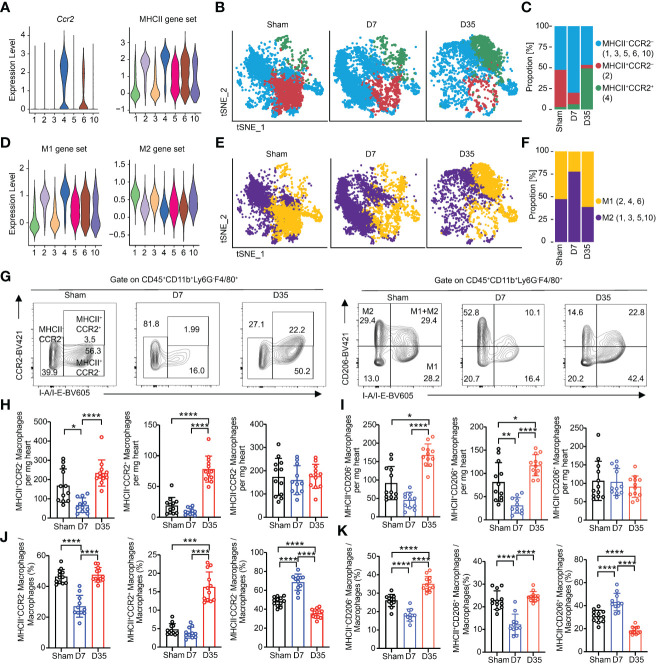
Myocardial macrophages are temporally polarized and dynamically changed after IR. **(A)** Violin plots exhibiting scores of Ccr2 and MHCII gene set on each macrophage subset. **(B)** Feature plots represented three subclusters based on Ccr2 and MHCII scores in Sham, D7 and D35 groups, respectively. MHCII-CCR2-: Cluster 1, 3, 5, 6, 10; MHCII+CCR2-: Cluster 2; MHCII+CCR2+: Cluster 4. **(C)** Proportion of three subclusters in panel **(B)** in three groups. **(D)** Violin plots exhibiting scores of M1 and M2 gene sets on each macrophage subset. **(E)** Feature plots represented two subclusters based on M1 and M2 scores in Sham, D7 and D35 groups, respectively. M1: Cluster 2, 4, 6; M2: Cluster 1, 3, 5, 10. **(F)** Proportion of three subclusters in panel **(E)** in three groups. **(G)** Flow cytometry analysis determined the three macrophage subsets with CCR2 and I-A/I-B (label MHCII) antibodies in three groups (left). Flow cytometry analysis determined the three macrophage subsets with CD206 and I-A/I-B antibodies in three groups (right). M1: CD206-I-A/I-B+, M2: CD206+I-A/I-B-; M1+M2: CD206+I-A/I-B+. **(H, I)** Numbers of indicated macrophage subsets in three groups by flow cytometry analysis. MHCII-CCR2-^a^, MHCII+CCR2-^b^, MHCII+CCR2+^c^; MHCII-CD206+^b^, MHCII+CD206-^b^, MHCII+CD206+^c^. **(J, K)** Proportions of indicated macrophage subsets in three groups by flow cytometry analysis. MHCII-CCR2-^a^, MHCII+CCR2-^a^, MHCII+CCR2+^b^; MHCII-CD206+^a^, MHCII+CD206+^a^, MHCII+CD206-^a^. Sham: n=12; D7: n=11; D35: n=12. Flow cytometry analysis data were pooled from three independent experiments. MHCII, major histocompatibility complex II.”a”: Ordinary one-way ANOVA test; “b”: Kruskal-Wallis test; “c”: Welch ANOVA test. *P<0.05, **P<0.01, ***P<0.001 and ****P<0.0001.

We sought to analyze the changes based on the expression of Ccr2 and MHCII or M1 and M2 marker genes over time. The expression of genes enrolled in the MHCII molecular score set were shown as a bubble chart (**Supplementary Figure S4A**). Feature plot showed the expressed genes based on MHCII gene sets (**Supplementary Figure S4C**). Ccr2 expression and MHCII scores of each cluster were shown as Violin plots (**Figure 5A**). Therefore, we classified all macrophages into three major groups: MHCII-CCR2- (1, 3, 5, 6, 10), MHCII+CCR2- (2), and MHCII+CCR2+ (4) (**Figure 5B**). The proportion of MHCII-CCR2- cells increased on Day 7 and decreased on Day 35, and the proportion of MHCII+CCR2+ cells appeared to increase on Day 35 (**Figure 5C**). Obviously, MHCII+ macrophages are the major cell type on Day 35 and might play an important role in APP. CCR2+ macrophages, which are considered to originate from monocytes, have an inflammatory phenotype.

M1 and M2 subtypes could still help us to understand the function of macrophages according to the proinflammatory role of the M1 type and the anti-inflammatory role of the M2 type in some other cardiac disease models (20, 54, 55). We found that Cluster 1 expressed Mcr1, Cd163 and Stab 1, which are classic M2 marker genes, and Cluster 4 expressed Il1b, which was considered as M1 marker gene (**Supplementary Figure S4B**). Then, we referred to the lists of M1/M2 gene scores from Yunfan Sun et al. (**Supplementary Table 2**) (38). Feature plot showed the expressed genes based on M1/M2 gene sets (**Supplementary Figure S4D**). Clusters 2, 4, and 6 were defined as M1-like macrophages, and Clusters 1, 3, 5, and 10 were considered as M2-like macrophages (**Figure 5D**). On Day 7, M2-like macrophages were the main cell type, and M1-like macrophages became the main type on Day 35 (**Figures 5E, F**).

We performed flow cytometry analysis experiments to verify the polarization with respect to MHCII/CCR2 and M1/M2. The gate for all macrophage subsets was based on the gate of total macrophages. We stained macrophages with CCR2-BV421 and I-A/I-E-BV605, or with CD206-BV421 and I-A/I-E-BV605 (M1-like: MHCII+CD206-; M2-like: MHCII-CD206+; M1+M2-like: MHCII+CD206+) (15, 56, 57) (**Figure 5G**). fluorescence minus one (FMO)-CCR2 and FMO-CD206 are shown in **Supplementary Figures S4E**, **S4F**.

First, we compared the absolute numbers of each subtype in heart tissues. The results showed that the number of MHCII+CCR2- macrophages decreased on Day 7 and increased on Day 35. MHCII+CCR2+ macrophages greatly increased on Day 35, possibly indicating infiltration of inflammatory monocytes into the heart tissues. Notably, MHCII-CCR2- cells maintained almost equal numbers among the three groups (**Figure 5H**). Meanwhile, the number of M1-like cells decreased on Day 7 and significantly increased on Day 35; the number of M2-like cells remained nearly constant; the number of M1+M2-like macrophages shared almost the same trends with M1 cells (**Figure 5I**).

Next, the percentages of each subtype were compared among groups. The percentage of MHCII+CCR2- cells obviously decreased on Day 7 and increased to sham level on Day 35; MHCII-CCR2- cells showed the opposite results to MHCII+CCR2- at these two-time points. On Day 35, the proportion of MHCII+CCR2+ cells increased significantly which was consistent with the absolute number results (**Figure 5J**). The percentage of M2-like cells increased on Day 7, while the percentage of M1-like cells increased on Day 35. M1+M2-like macrophages obviously decreased on Day 7 and returned to sham proportion on Day 35. (**Figure 5K**).

In conclusion, single-cell RNA sequencing and flow cytometry analysis both showed that the proportions of MHCII-CCR2- and M2-like macrophages increased on Day 7 and decreased on Day 35, and the proportions of MHCII+CCR2+ and M1 macrophages increased significantly on Day 35. Interestingly, flow cytometry analysis demonstrated the absolute number of MHCII-CCR2- and M2-like macrophages remained almost constant, while MHCII+ macrophages slightly decreased on Day 7 and largely expanded on Day 35.

### Different roles of macrophages on Day 7 and Day 35 after cardiac IR

In our recent study, we observed changes in cardiac macrophage subsets, which implied potentially different functions of macrophages at specific stages after cardiac IR. We aimed to understand how macrophages function at different stages following IR by comparing gene expressions among three groups.

**Figure 6:**
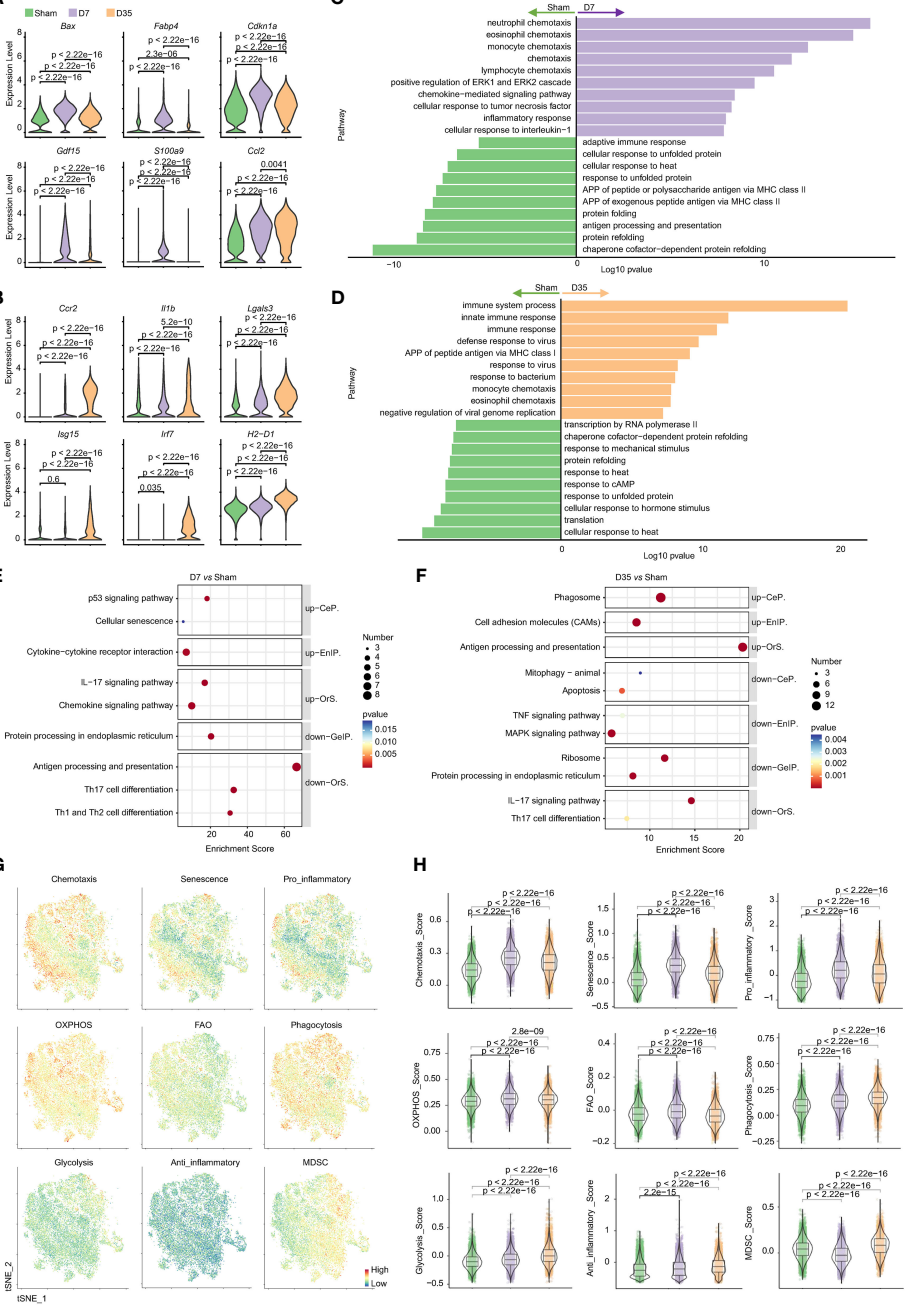
Characteristic genes and biological functions of macrophages in two stages post cardiac IR. **(A, B)** Violin plots presented selected characteristic genes expressed higher in D7 group **(A)** and D35 group **(B)**. **(C, D)** GO pathway analysis of differentially genes on biological process: D7 vs Sham **(C)**, D35 vs Sham **(D)**. **(E, F)** KEGG pathway analysis of differentially genes: D7 vs Sham group **(E)**, D35 vs Sham **(F)**. up, up-regulated pathways in D7 or D35 group; down, down-regulated pathways in D7 or D35 group. **(G)** Feature plots determining the levels of phagocytosis, chemotaxis, glycolysis, OXPHOS, anti-inflammatory, pro-inflammatory, senescence, FAO, MDSC in total macrophages (three groups included). **(H)** Violin plots presented scores comparisons among the three groups. GO, Gene Ontology; KEGG, the Kyoto Encyclopedia of Genes and Genomes; CeP, Cellular Processes; EnIP, Environmental Information Processing; Ors, Organismal Systems; GeIP, Genetic Information Processing. APP, antigen processing and presentation; OXPHOS, oxidative phosphorylation; FAO, fatty acid oxidation; MDSC, myeloid-derived suppressor cells.

The top 20 DEGs for pairwise comparisons are shown in volcano plots (**Supplementary Figures S5A**, **B**). Compared to the Sham group, chemokines (Ccl2, Ccl7, Ccl8) and inflammatory myeloid-related genes (S100a4, S100a6, S100a9) were upregulated, but heat shock protein genes were downregulated in two IR groups. In special, the 7-day group overexpressed proapoptotic genes (Cdkn1a, Bax), lipid metabolism-related genes (Fabp4) and stress-responsive cytokines (Gdf15). And the 35-day group strongly overexpressed inflammatory genes (Ccr2, Lgals3, Il1b) and interferon-stimulated genes (Ifit3, Isg15, Irf7). Moreover, MHCII genes (H2-T23, H2-D1, H2-K1) were upregulated on Day 35 and decreased on Day 7, in line with the flow cytometry analysis. Typical genes are shown in **Figures 6A, B**. The differences in each gene among the three groups were significant, which probably indicated the diversity and heterogeneity of macrophages at these two time points after IR.

GO biological process analysis (**Figures 6C, D**) and KEGG enrichment analysis (**Figures 6E, F**) indicated that 7-day group was enriched with chemokine signaling pathway and inflammatory pathway (ERK1 and ERK2 cascades, cellular response to TNF and interleukin-1, p53 signaling pathway). For D35 vs. Sham, APP, defense responses to viruses and bacteria, phagosomes and CAMs were enriched in 35-day group. In contrast, two IR groups both exhibited lower responses to heat and unfolded proteins and decreased levels of protein folding and refolding. In addition, 7-day group was associated with decreased APP levels and the adaptive immune response; 35-day group was characterized by a lower response to cAMP, mechanical stimulus, hormone stimulus, and a lower level of transcription and translation. Of note, an increase in cellular senescence was observed in 7-day group, and a decrease in mitophagy was observed in the 35-day group, although very few genes were enriched. In addition, GO analysis on molecular function showed that ATPase activity and ATP binding were obviously decreased in both IR groups compared with the sham group, which indicated mitochondrial dysfunction (**Supplementary Figure S5C**).

The comparisons between D35 and D7 are shown in **Supplementary Figures S5D-F**. It is worth noting that “intrinsic apoptotic signaling pathway in response to DNA damage by p53 class mediator” and “activation of cysteine-type endopeptidase activity involved in apoptotic process” were downregulated in 35-day group, which means a relative apoptotic state of macrophages on Day 7 and a relatively reduced apoptotic state or a repaired state of macrophages on Day 35. In addition, “positive regulation of inflammatory response” and “neutrophil chemotaxis” were downregulated in the 35-day group, implying reduced inflammation. The results were in line with the flow cytometry analysis showing that neutrophils infiltrated on Day 7 and recovered to normal levels on Day 35. Moreover, “regulation of translation”, “translation” and “ribosomal large or small subunit assembly” were all downregulated, indicating dysfunction of protein synthesis on Day 35.

Next, to further understand the biological states of total macrophages in each group, we used gene scores to compare the differences in chemotaxis, senescence, pro-inflammation, anti-inflammation, energy metabolism, liposome metabolism, phagocytosis and MDSCs (**Figures 6G, H**). The results showed that 7-day group had higher scores of chemotaxis, senescence, pro-inflammation, OXPHOS and FAO. In contrast, phagocytosis, glycolysis, anti-inflammation, and MDSC scores were consistently increased in 35-day group.

Moreover, macrophages make up the vast majority of the heart’s immune cells, accounting for more than 70%. Thus, we tried to test the typical genes at the two time points in immune cells to verify the changes in macrophages. RT-PCR results showed that Cdkn1a, Gdf15, Bax were significantly increased on Day 7, and Lgals3, Isg15, Irf7, Ccr2 were obviously increased on Day 35 (**Supplementary Figure 6S**), which demonstrated the reliability of single cell sequencing results.

Lastly, SCENIC analysis was performed by groups. Heat maps of RAS activity of regulons showed that AP-1 family members (Jun, Junb, Jund) and Atf3, were highly expressed in Sham group and gradually decreased by time dependent manner in IR groups. Interferon-related TFs (Stat1, Stat2, Irf5, Irf7), Fosl2 and Etv3 were strongly activated in 35-day group. 7-day group was shown low activity of most TFs. (**Supplementary Figure S7A**) RSS-specific heat maps of regulons identified that Atf3, Jun, Jund, Ets1, Ets2 may be specifically related to macrophages in three groups and Dbp was specific related to 7-day group cells (**Supplementary Figure S7B**). CSI correlation clustering heat map of Regulon module demonstrated that module 1 which contained TFs such as Irf1, Cebpg, Junb, Atf3, Jun, Jund was with the highest activity (**Supplementary Figures S7C, D**).

In conclusion, 7-day group was characterized by a lower ability of APP processing with a probably senescent state accompanied by a stronger inflammatory response. 35-day group macrophages were characterized with lower levels of transcription, increased processing of APP, phagocytosis and cell adhesion but reduced inflammation. SCENIC analysis showed AP-1 family members are strongly associated with changes in the phenotype of cardiac macrophages in our model.

## Discussion

In our study, we noticed a temporary decline in heart function accompanied by changes of cardiac macrophages in early stage after IR. Based on the vital role of macrophages in cardiac function, we speculate that cardiac macrophages may play an important role in IR-induced heart injury. Therefore, we identified subtypes of cardiac macrophages and described their dynamics and functions by single-cell RNA sequencing on cardiac immune cells. It’s noteworthy that ours is the first study focusing on the immune status of macrophages at an early stage in cardiac IR model mice.

Diastolic dysfunction was observed one week after heart IR in rat models (10, 58). We also found a similar dysfunction initially, and accompanied by a decrease of macrophages on Day 7. However, mice survived this period and eventually recovered heart function, possibly due to the accumulation of macrophages thereafter. Although previous studies reported an increase in cardiac macrophages after IR, none had detailed the changes or potential functions of these cells.

Previous studies have suggested that murine myocardium consists of two kinds of CCR2- RTMs (Lyve1+MHCII- and Lyve1-MHCII+ macrophages), which are derived from fetal liver and yolk-sac-derived progenitor cells and do not depend on peripheral monocyte supply but rather self-renew (15, 16, 59). Removing CCR2- cell populations can affect cardiac conduction and diastolic function- (60). Cardiac M2 macrophages (MHCII-CD206+) were considered to have anti-inflammatory and reparative phenotype (15) In our study, Cluster 1 and 2 were two classical CCR2- RTMs, making up most of the stable heart macrophages. Cluster 1 expressed more M2 marker genes with no MHCII genes expression, which may be correspond to MHCII-CCR2- and MHCII-CD206+ macrophages in our flow cytometry, indicating its potential cardioprotective and anti-inflammatory function. Cluster 2 highly expressed MHCII molecules and lacked M2 marker genes, suggesting its APP ability with proinflammatory function. We found the proportion of Cluster 1 remained unchanged by single-cell RNA sequencing, and the absolute number of MHCII-CCR2- and MHCII-CD206+ macrophages remained unchanged over time from flow cytometry results. Therefore, we suspect that mice survived and heart function recovered on Day 35 probably due to the long-lived and cardioprotective Cluster 1. In contrast, Cluster 2 progressively decreased over time and nearly vanished on Day 35. Similar vanishing was observed in MI models (21, 61, 62). We suspect Cluster 2 might transform or die, but more research is needed to confirm this.

Notably, we identified two macrophage subtypes induced by IR: Cluster 3 on Day 7 and Cluster 4 on Day 35. Except for the similar gene signatures (MHCII-CCR2-) and similar trajectory of differentiation with Cluster 1, Cluster 3 showed attenuated expression of classical RTM genes and M2-related genes, but had a higher response to LPS with greater chemotaxis, higher inflammation, and senescent status. We hypothesize that Cluster 3 was transforming to an inflammatory subtype maybe Cluster 4. This explained the phenomenon that Cluster 3 expanded and then receded over time. Moreover, Cluster 4 highly expressed Ccr2 and MHCII genes but lacked M2-related genes, suggesting an M1-like phenotype with stronger APP, improved phagocytosis ability and strong pro-inflammatory function. The trajectory analyses suggested that Cluster 4 probably originated from monocytes. The flow cytometry analysis showed that Ly6C^high^ monocytes increased significantly on Day 35, which again verified the possibility of peripheral proinflammatory monocytes entering cardiac tissue to proliferate and supplement CCR2 macrophages at this stage. In addition, MI provokes activation of an IRF3–interferon axis in a distinct population of cardiac macrophages, and IRF3 and the type I IFN response were considered as potential therapeutic targets for protection (63). Cluster 4, which highly expressed Isg15, was confirmed by immunofluorescent staining. It appeared later in our model than it did in the MI mouse model (23), which might be the therapeutic target cells in this stage.

Next, we observed polarization and dynamic changes of macrophages based on M1/M2 and Ccr2 and MHCII genes expression over time by single-cell RNA sequencing, indicating a protective or anti-inflammatory role on Day 7 and an injury or inflammatory role on Day 35. We confirmed the proportion changes of these subtypes by flow cytometry. But interestingly, we found a sharp decrease in number of most subtypes except MHCII-CCR2- and MHCII-CD206+ (M2-like) macrophages on Day 7. The percentage of MHCII-CCR2- and M2-like macrophages increased on Day 7, probably because the number of these two types remained unchanged and the other subtypes decreased. Similarly, the proportions of MHCII+ and M1-like macrophages increased because of their expansion in numbers. When performed gene score analysis and pathway enrichment analysis, we found that the overall characteristics of macrophages on Day 7 were elevated inflammatory cytokines and chemotaxis, probably due to the important role of inflammatory Cluster 3 (MHCII-CCR2-CD206+). Besides, stronger immune response, increased phagosome and CAMs, elevated APP with reduced inflammation were shown on Day 35. The inconsistency here once again tells us about the heterogeneity and diversity of macrophages and that anti-inflammatory and pro-inflammatory factors cannot be judged by a single criterion.

Compared with widely studied MI mice model, macrophages in our model exhibited different phenotypes and characteristics (15, 16). First, macrophages exhibited a polarization from M2(day 7) to M1(day 35) phenotype, and macrophages with high M2-labeled expression had a stronger inflammatory phenotype. Second, the number of macrophages showed a transient decline initially, showing an aging state of increased apoptosis, increased inflammation, and enhanced chemotactic ability, which is unique and not found in MI or other cardiac injury models. Third, MHCII+CCR2+ macrophages accompanied by inflammatory monocyte infiltration occurred later, and the number was not as large as that in other cardiac disease models, representing a delayed and relatively mild inflammation. Fourth, despite the decrease of chemokines and the reduction of inflammation over time, number of macrophages increased accompanied by elevated inflammatory interferon-related pathways although heart function is back to normal. Previous study focused on radiated human arteries showed sustained inflammation due to nuclear factor-kappa B activation (64). In our model, whether there is persistent inflammation in the heart, and what the phenotype of macrophage is after 35 days, needs further study.

SCENIC analysis showed a decline of transcription factors Aft3, Jun, Junb, Jund over time after IR. ATF3 was reported significantly down-regulated in hematopoietic stem cells after exposure to irradiation which was in line with our results (65). Besides, ATF3 could protect cells from UV-induced apoptosis in a p53-dependent manner (66). JunD was proved to protect cells from p53-dependent senescence and apoptosis (67). Therefore, Aft3 and Jund might be potential targets to protect macrophages from apoptosis in our model. Moreover, transcription factor Dbp was upregulated on Day 7, which was reported as a key age-associated regulator in cerebral ischaemia (68) and involved in CCL2 activation in mesangial cells (69). We suspect that downregulated Dbp could potentially protect cells from senescence and apoptosis and reduced Ccl2 related inflammation.

Radiation could make mitochondrial DNA double-strand breaks (mtDSBs) which are toxic lesions that alter mitochondrial function (70). After the formation of mtDSBs, herniation (71) mediated by BAX and BAK releases mitochondrial RNA into the cytoplasm and triggers a type-I interferon response that involves the phosphorylation of STAT1 and activation of interferon-stimulated genes. Treatment of mtDNA-proficient cells with 20 Gy irradiation resulted in a reduction of approximately 40% in mitochondrial genomes, while STAT1 signalling and ISGs induction were diminished when cells lacking mtDNA. In our study, similar gene expression changes were observed. Mitochondrial dysfunction was showed in two IR groups according to pathway enrichment analysis. Apoptosis-related gene “Bax”, which is considered as mitochondrial apoptotic effector, was highly expressed on Day 7. Then, strong interferon response was identified on Day 35. In addition, “intrinsic apoptotic signaling pathway in response to DNA damage by p53 class mediator” and “p53 signaling pathway” were upregulated on Day 7 comparing with Day 35. Hence, we suspect an apoptosis state on Day 7 and reduced apoptosis state but increased interferon state on Day 35 which were associated with p53 signaling pathway. But the process or transformation from apoptosis to interferon should be further studied in the future.

The limitations of this study are as follows: First, a single dose of 20 Gy, which is not clinically relevant, has been widely used in radiation-induced heart injury animal models (28, 30, 31). But the results should be cautiously extrapolated to clinical practice. Second, mouse and human macrophages exhibit different signatures (72), so it is difficult to transpose experimental data obtained in our mouse model to humans. Third, radiation during small animal tests might affect the lungs, impacting heart function shortly after (73). Changes in lung and heart weight suggest potential heart function decline due to lung issues. Fourth, the study describes macrophage traits at two-time points after radiation but doesn’t explain how these cells transform. Fifth, we identified two kinds of stage specific macrophages after radiation, but how they change from one type to the other is unclear. Sixth, it’s suggested that certain macrophages are important, but it’s not tested if removing them could help the heart long-term. Last, the study doesn’t look at macrophage types beyond 35 days after radiation in the heart.

## Conclusions

We established a mouse model of IR-induced heart injury to investigate the early changes in cardiac function and to explore the role of cardiac macrophages in this process. To our knowledge, this is the first study to characterize the diversity, features, and evolution of cardiac macrophages during the early stages in a mouse model. First, we proved that radiation-induced heart injury occurs one week after IR and is accompanied by changes in the immune microenvironment, especially macrophages. Second, we identified 7 clusters of macrophages by single-cell RNA sequencing and found two kinds of stage specific macrophages on Day 7 and Day 35. Third, we observed cardiac macrophages polarized over these two-time points based on M1/M2 and CCR2/MHCII expression. Lastly, pathway enrichment analyses suggested that macrophages on Day 7 were characterized by an inflammatory senescent phenotype with enhanced chemotaxis and inflammatory factors, while macrophages on Day 35 showed enhanced phagocytosis, which was associated with interferon-related pathways. This study might provide insight into explore early intervention strategies by targeting or regulating the function and phenotype of cardiac macrophages to reduce heart injury.

## Data availability statement

The data presented in the study are deposited in the Sequence Read Archive Database repository, accession number PRJNA1088304.

## Ethics statement

The animal study was approved by Shanghai Jiao Tong University School of Medicine Institutional Animal Care and Use Committee. The study was conducted in accordance with the local legislation and institutional requirements.

## Author contributions

CC: Writing – original draft, Writing – review & editing. RW: Writing – review & editing. SW: Writing – review & editing. LZ: Writing – review & editing, Software, Data curation. PC: Writing – review & editing, Formal analysis. SL: Writing – review & editing, Visualization, Funding acquisition. QZ: Writing – review & editing, Validation, Funding acquisition. HL: Writing – review & editing, Methodology, Investigation. YL: Writing – review & editing, Project administration. ML: Writing – review & editing, Supervision, Funding acquisition. LC: Writing – review & editing, Supervision, Funding acquisition. JC: Writing – review & editing, Resources, Conceptualization, Funding acquisition.
