# Supplementary material for: Elucidating the changes in the heterogeneity and function of radiation-induced cardiac macrophages using single-cell RNA sequencing
Source: Front Immunol. 2024 Mar 27;15:1363278. doi: 10.3389/fimmu.2024.1363278 (PMC11004337; doi:10.3389/fimmu.2024.1363278)
Supplement: Supplementary file 1 [file DataSheet_1.docx]

**Figure S1. Immunohistochemical γH2AX staining of heart tissue, echocardiographic analysis of cardiac function and flow cytometry gating scheme used to identify live cardiac immune cells.** A. Representative images of γH2AX immunofluorescent staining of heart tissue: sham and IR one hour later. Each image was from an individual mouse heart; γH2AX+ broken DNA (Red), DAPI+ nucleus (Blue). (n=3 in each group). B. Other parameters of systolic function by M-mode: left ventricular ejection fraction (LVEF), left ventricular fractional shortening (LVFS), left ventricular internal diameter in diastole (LVIDd), left ventricular internal diameter in systole (LVIDs), left ventricular end-diastolic volume (LVEDV), left ventricular end-systolic volume (LVESV), left ventricular posterior wall thickness in diastole (LVPWd) and left ventricular posterior wall thickness in systole (LVPWs). C. Other parameters of diastolic function by blood flow doppler and tissue doppler: the peak early diastolic mitral annular velocity (e'), the peak late diastolic mitral annular velocity (a’), aortic ejection time (AET), isovolumic contraction times (IVCT), isovolumic relaxation (IVRT), the E/A ratio, the E/e’ ratio and the e’/a’ ratio. D. Flow cytometry gating scheme used to identify live cardiac immune cells. LVPWd: Kruskal-Wallis test. The others: Ordinary one-way ANOVA test.

**Figure S2. Single cell RNA sequencing identifying CD45+ immune cells in mice heart tissues under IR or sham treatment.** A. Live cardiac CD45+ immune cells in each group were separated by magnetic beads ion for 10× genomics single-cell RNA sequencing analyses. Six individual hearts were digested and mixed in each group as one sample. B. t-SNE dimensionality reduction analysis identified 5 major cell lineages including 26855 individual cells in total. C. The proportion of each cell lineage in Sham, D7 and D35 group. The number of B cells, Basophils, Monocyte lineage, Neutrophils, and T cells. D. Heatmap showing top 10 different expression marker genes for B cells, Basophils, Monocyte lineage, Neutrophils, and T cells. t-SNE, t-distributed stochastic neighbor embedding.

**Figure S3. Supplementary KEGG pathway analysis of DEGs and immunofluorescent staining of ISG15+ macrophages.** A. KEGG pathway analysis between Cluster 3 and Cluster 1. B. KEGG pathway analysis between Cluster 4 and Cluster 2. Up, up-regulated pathways in Cluster 3 or Cluster 4; down, down-regulated pathways in Cluster 3 or Cluster 4; CeP, Cellular Processes; EnIP, Environmental Information Processing; Ors, Organismal Systems; GeIP, Genetic Information Processing; KEGG, the Kyoto Encyclopedia of Genes and Genomes; DEGs, differentially genes.

**Figure S4.** **Expression of interesting genes or gene sets by single cell RNA sequencing, and fluorescence minus one control in flow cytometry analysis.** A. Dot plot exhibiting expression of genes in MHCII gene set in each macrophage subset. B. Dot plot exhibiting expression of representative M1 (Il1b) and M2 (Mrc1, Cd163 and Stab1) genes in each macrophage subset. C-D. Feature plots showing the expressed genes based on MHCII, M1 and M2 gene sets. E-F. FMO controls (bottom) of CCR2 (E) and CD206 (F) were included for precise gating. MHCII，major histocompatibility complex II；FMO, fluorescence minus one.

**Figure S5. Supplementary materials on DEGs and pathway analysis of macrophages in two stages post cardiac IR.** A-B. Volcano plots showing DEGs between D7 and Sham group (A), and between D35 and Sham group (B). Genes in red represented up-regulated genes in D7 (or D35) group compared with Sham group. Genes in blue represented down-regulated genes in D7 (or D35) group compared with Sham group. C. Top 10 down-regulated GO terms on molecular function in D7 (or D35) group compared with Sham group. D. Volcano plots showing DEGs between D35 and D7 group. Genes in red represented up-regulated genes in D35 group compared with D7 group. Genes in blue represented down-regulated genes in D35 group compared with D7 group. E. GO pathway analysis of DEGs on biological process between D35 and D7 group. F. KEGG pathway analysis of DEGs between D35 and D7 group. DEGs, differentially expressed genes; GO, Gene Ontology; KEGG, the Kyoto Encyclopedia of Genes and Genomes; APP，antigen processing and presentation.

**Figure 6S. Quantitative analysis of transcriptional expression of genes of interest in cardiac immune cells by qRT-PCR.** A. 7-day typical genes, B. 35-day typical genes. After single-cell RNA sequencing, the total RNA of the remaining immune cells from each sample was extracted. We made three duplicate holes for each sample, and β-actin was used as an internal control. Ordinary one-way ANOVA test was used for all genes. **P*<0.05, ****P*<0.001 and *****P*<0.0001.

**Figure 7S.** **Single-cell regulatory network inference and clustering (SCENIC)** **analysis by groups.** A. Heat maps of RAS activity of regulons in each group; A color changing from blue to red indicates a RAS activity score from low to high, and a higher RAS score indicates a stronger activity of regulon in that cell population. B. RSS-specific heat maps of regulons in each group; The color changing from blue to red indicates a low to high RSS specificity score, and a higher RSS score indicates a stronger specificity of regulon in that cell population. C. CSI correlation clustering heat map of Regulon module; D. Heat maps of activity of CSI correlation modules. The color from blue to yellow indicates the CSI module activity from low to high. RAS, regulon activity score; RSS, regulon specificity score; CSI, connection specificity index.
