# Supplementary figures and images for: Elucidating the changes in the heterogeneity and function of radiation-induced cardiac macrophages using single-cell RNA sequencing
Source: Front Immunol. 2024 Mar 27;15:1363278. doi: 10.3389/fimmu.2024.1363278 (PMC11004337; doi:10.3389/fimmu.2024.1363278)

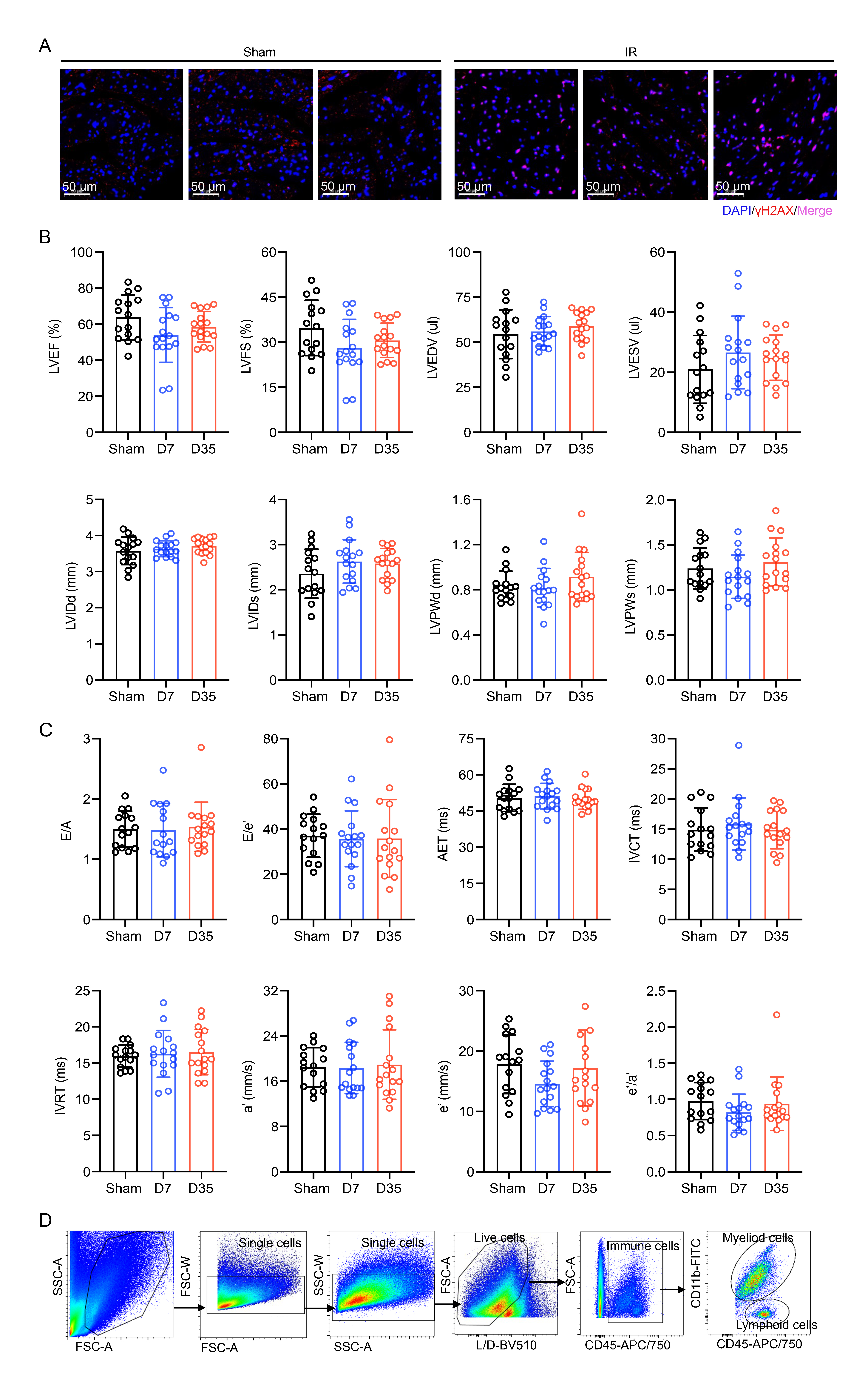

Supplement: Supplementary file 2 [file Image_1.tif]

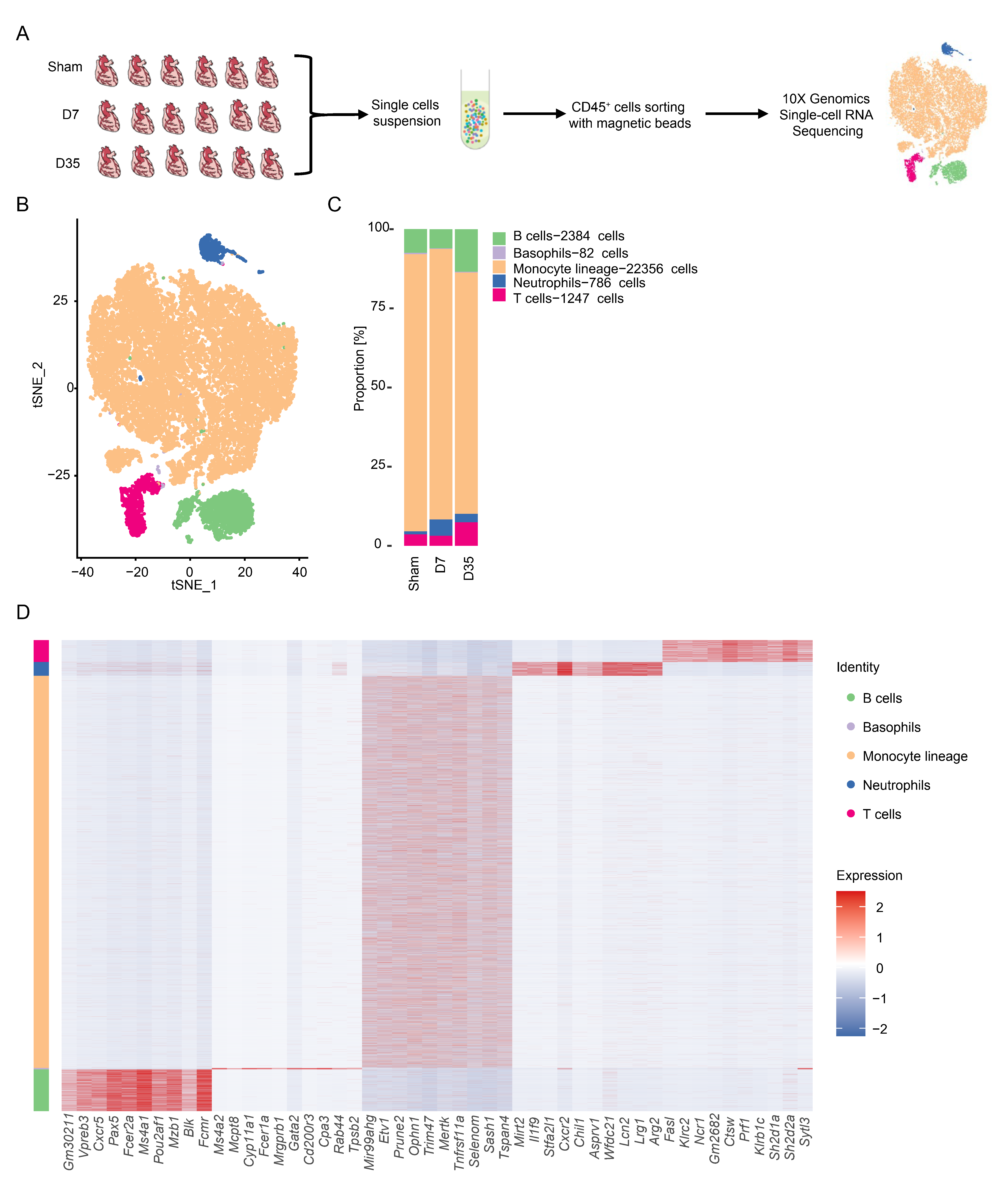

Supplement: Supplementary file 3 [file Image_2.tif]

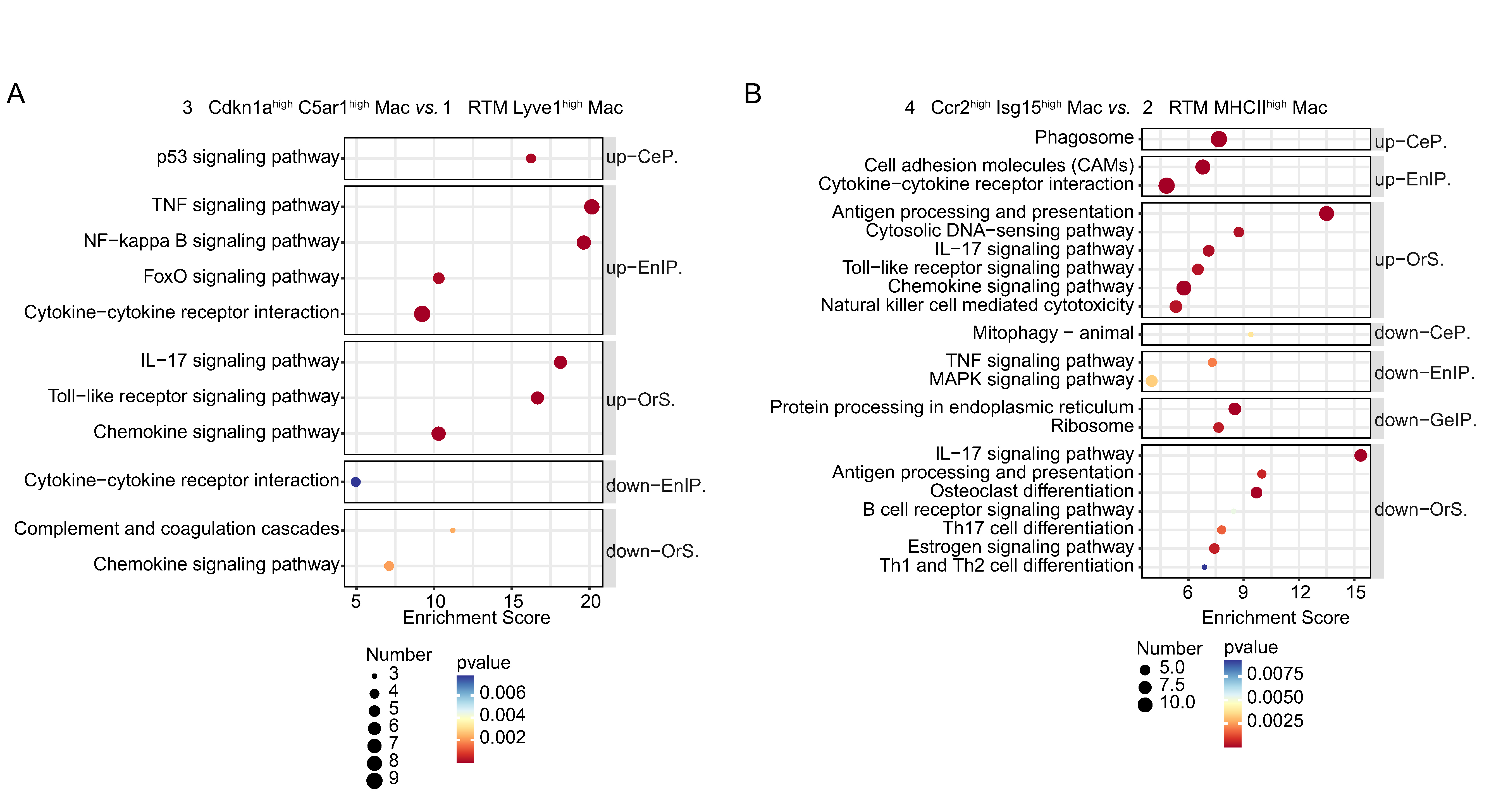

Supplement: Supplementary file 4 [file Image_3.tif]

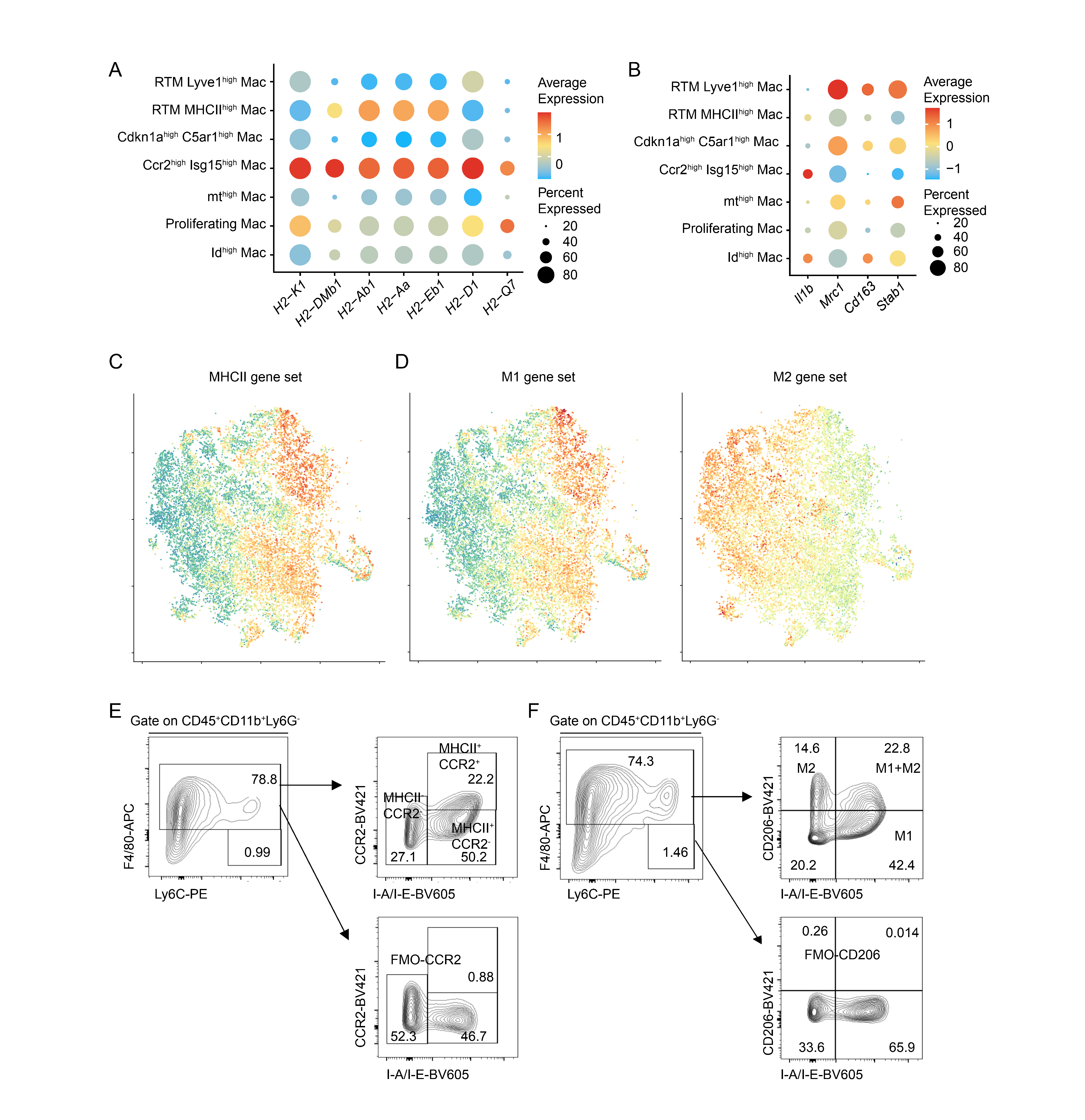

Supplement: Supplementary file 5 [file Image_4.tif]

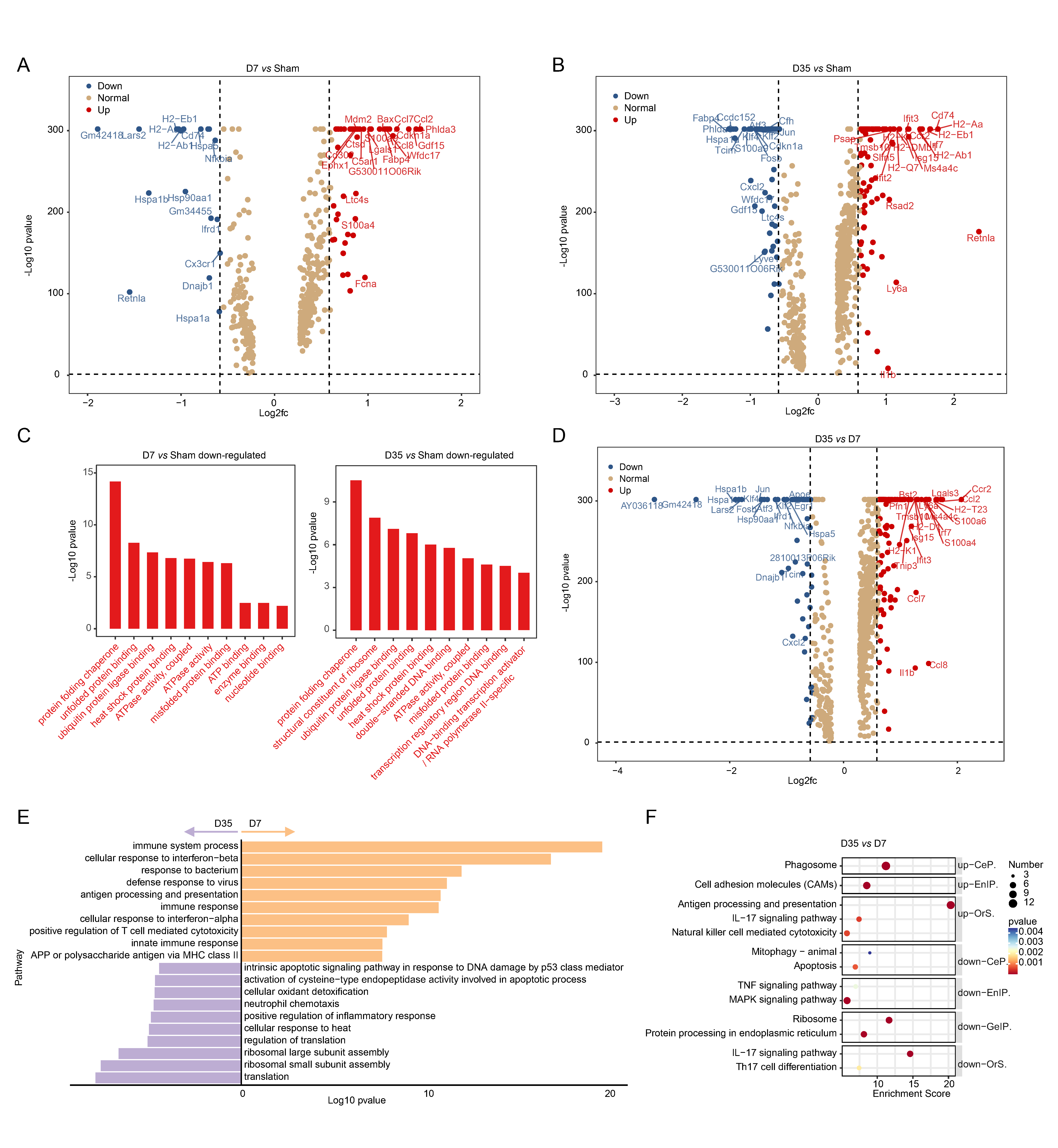

Supplement: Supplementary file 6 [file Image_5.tif]

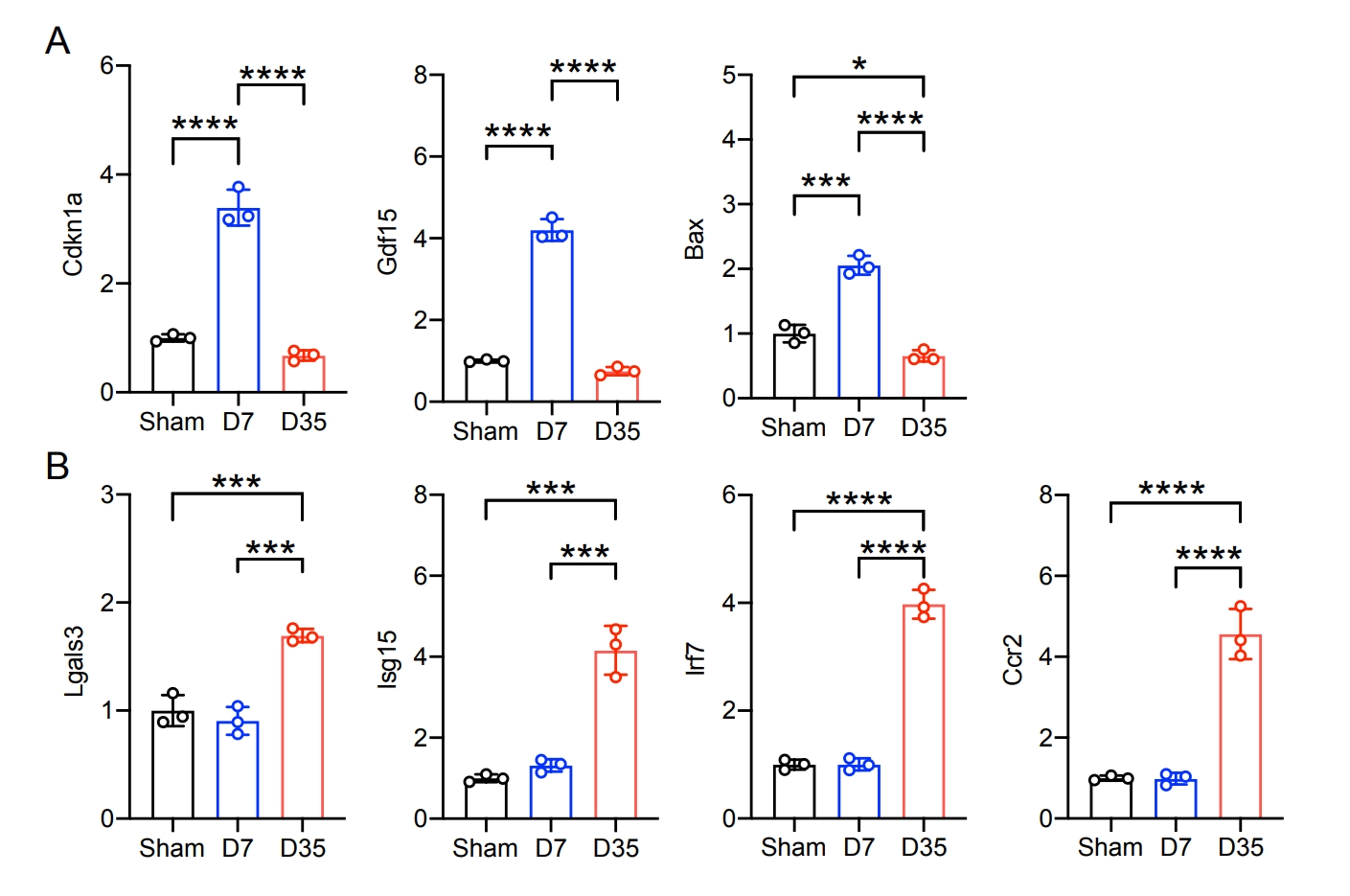

Supplement: Supplementary file 7 [file Image_6.jpeg]

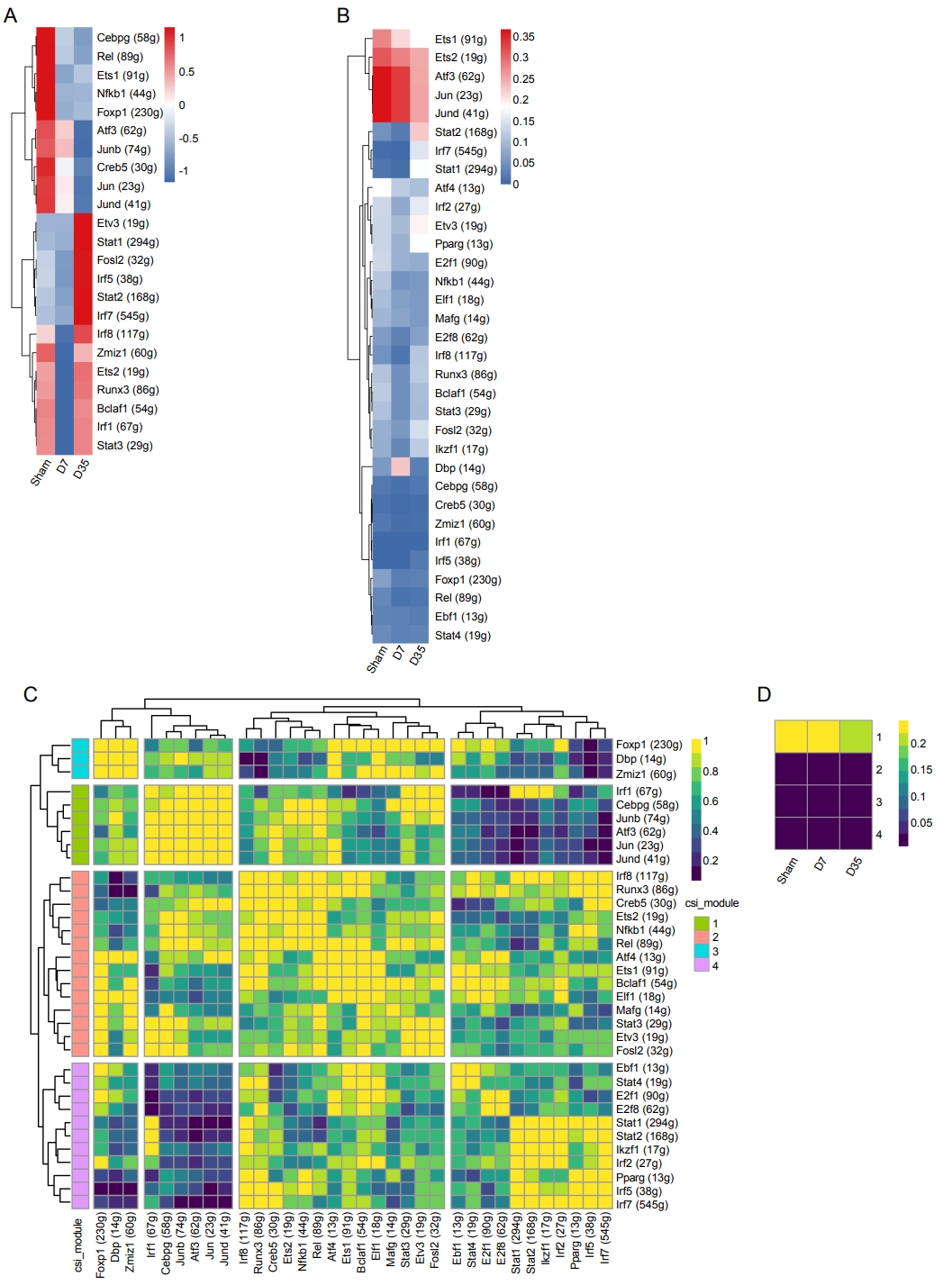

Supplement: Supplementary file 8 [file Image_7.jpeg]
